# Independent Associations of Fasting Insulin, Glucose, and Glycated Haemoglobin with Stroke and Coronary Heart Disease in Older Women

**DOI:** 10.1371/journal.pmed.0040263

**Published:** 2007-08-28

**Authors:** Debbie A Lawlor, Abigail Fraser, Shah Ebrahim, George Davey Smith

**Affiliations:** 1 Department of Social Medicine, University of Bristol, Bristol, United Kingdom; 2 Department of Epidemiology and Population Health, London School of Hygiene and Tropical Medicine, London, United Kingdom; The George Institute for International Health, Australia

## Abstract

**Background:**

Evidence suggests that variations in fasting glucose and insulin amongst those without frank type 2 diabetes mellitus are important determinants of cardiovascular disease. However, the relative importance of variations in fasting insulin, glucose, and glycated haemoglobin as risk factors for cardiovascular disease in women without diabetes is unclear. Our aim was to determine the independent associations of fasting insulin, glucose, and glycated haemoglobin with coronary heart disease and stroke in older women.

**Methods and Findings:**

We undertook a prospective cohort study of 3,246 British women aged 60–79 y, all of whom were free of baseline coronary heart disease, stroke, and diabetes, and all of whom had fasting glucose levels below 7 mmol/l. Fasting insulin and homeostasis model assessment for insulin sensitivity (HOMA-S) were linearly associated with a combined outcome of coronary heart disease or stroke (*n* = 219 events), but there was no association of fasting glucose or glycated haemoglobin with these outcomes. Results were similar for coronary heart disease and stroke as separate outcomes. The age, life-course socioeconomic position, smoking, and physical activity adjusted hazard ratio for a combined outcome of incident coronary heart disease or stroke per one standard deviation of fasting insulin was 1.14 (95% CI 1.02–1.33). Additional adjustment for other components of metabolic syndrome, low-density lipoprotein cholesterol, fasting glucose, and glycated haemoglobin had little effect on this result.

**Conclusions:**

Our findings suggest that in women in the 60–79 y age range, insulin resistance, rather than insulin secretion or chronic hyperglycaemia, is a more important risk factor for coronary heart disease and stroke. Below currently used thresholds of fasting glucose for defining diabetes, neither fasting glucose nor glycated haemoglobin are associated with cardiovascular disease.

## Abstract 2

From a prospective study of women aged 60-79 years, Debbie Lawlor and colleagues conclude that insulin resistance is an important risk factor for coronary heart disease and stroke.

## Editors' Summary

**Background.:**

Narrowing of the vessels that take blood to the heart and brain is a common form of cardiovascular disease—i.e., a disorder of the heart and blood vessels. It is a major cause of illness and death. By starving the heart and brain of oxygen, this condition causes coronary heart disease (CHD; heart problems such as angina and heart attacks) and strokes. A major risk factor for CHD and strokes is diabetes, a common chronic disease characterized by high levels of sugar (glucose) in the blood. In people who don't have diabetes, the hormone insulin controls blood-sugar levels. Insulin, which is released by the pancreas after eating, “instructs” insulin-responsive muscle and fat cells to absorb the glucose (released from food) from the bloodstream. In the very early stages of type 2 diabetes (the commonest type of diabetes, also called “adult onset” or “noninsulin-dependent” diabetes”), muscle and fat cells become unresponsive to insulin, so blood-sugar levels increase. This is called “insulin resistance.” The pancreas responds by making more insulin. As a result, people with insulin resistance have high blood levels of both insulin (hyperinsulinemia) and glucose (hyperglycemia). Eventually, the insulin-producing cells in the pancreas start to malfunction, insulin secretion decreases, and type 2 diabetes is the result.

**Why Was This Study Done?:**

It is not yet clear whether it is insulin resistance or reduced insulin secretion that is responsible for the association between diabetes and cardiovascular disease. Physicians would like to know this information to help them to prevent CHD and strokes in their patients. There is evidence that variations in fasting glucose levels (blood glucose measured more than 8 h after eating), which provide an indication of how well pancreatic cells are producing insulin, and in fasting insulin levels, which provide an indication of insulin resistance, determine cardiovascular disease risk among people without type 2 diabetes, but the relative importance of these risk factors is unclear. In this study, the researchers have investigated whether markers of insulin resistance (fasting hyperinsulinemia) and of altered insulin secretion (fasting hyperglycemia, and increased glycated hemoglobin, which indicates how much sugar has been in the blood over the past few months) are associated with CHD and strokes in elderly women without diabetes. Their aim is to gain new insights into how diabetes affects cardiovascular disease risk.

**What Did the Researchers Do and Find?:**

The researchers measured glucose, insulin, and glycated hemoglobulin in fasting blood samples taken from about 3,000 women aged 60–79 y when they enrolled in the British Women's Heart and Health Study. None of the women had CHD at enrollment, none had had a stroke, none had diagnosed diabetes, and all had a fasting blood glucose below 7 mmol/l (a higher reading indicates diabetes). After monitoring the women for nearly 5 y for CHD and strokes, the researchers looked for statistical associations between the occurrence of cardiovascular disease and markers of insulin resistance and reduced insulin secretion. They found that fasting insulin levels, but not fasting glucose or glycated hemoglobin levels, were associated with CHD and stroke, even after allowing for other factors that affect cardiovascular disease risk such as smoking and physical activity. In other words, raised fasting insulin levels increased the women's risk of developing cardiovascular disease.

**What Do These Findings Mean?:**

These results indicate that in elderly women without diabetes, fasting insulin (a marker of insulin resistance) is a better predictor of future cardiovascular disease risk than fasting glucose or glycated hemoglobin (markers of reduced insulin secretion). This suggests that insulin resistance might be the main mechanism linking type 2 diabetes to CHD and stroke in elderly women. (Elderly women are known to run a high risk of developing these conditions, but they have been relatively neglected in previous studies of the risk factors for cardiovascular disease.) However, because relatively few women developed CHD during the study and even fewer had a stroke, this conclusion needs confirming in larger studies, preferably ones that include more rigorous tests of insulin resistance and secretion and also include women from more ethnic backgrounds than this study did. If the association between fasting insulin levels and cardiovascular disease risk is confirmed, therapeutic interventions or lifestyle interventions (for example, increased physical activity or weight loss) that prevent or reverse insulin resistance might reduce cardiovascular disease risk better than interventions that prevent chronic hyperglycemia.

**Additional Information.:**

Please access these Web sites via the online version of this summary at http://dx.doi.org/10.1371/journal.pmed.0040263.

- MedlinePlus encyclopedia page on coronary heart disease, stroke, and diabetes (in English and Spanish)
- Information for patients and caregivers from the US National Diabetes Information Clearinghouse on diabetes, including information on insulin resistance and on diabetes, heart disease, and stroke
- Information on the British Women's Heart and Health Study

## Introduction

Diabetes is known to be one of the most potent risk factors for coronary heart disease (CHD) and stroke in women and men [1–3], but the mechanisms for these associations are unclear. Insulin resistance is a precursor to glucose intolerance and type 2 diabetes mellitus. The progression from insulin resistance to impaired glucose tolerance (characterised predominantly by postprandial hyperglycaemia) occurs as a result of early pancreatic β-cell dysfunction [4,5]. With worsening β-cell dysfunction this leads to overt type 2 diabetes [4,5]. Once diabetes has occurred there is no further marked deterioration in insulin resistance; instead, poor insulin secretion (β-cell dysfunction) becomes the main pathological process [6,7]. Thus, the association of type 2 diabetes with cardiovascular disease may reflect determinants and consequences (including hyperinsulinaemia) of insulin resistance, or determinants and consequences (including hyperglycaemia) of reduced insulin secretion, or a combination of these processes. Exploring the relative associations of hyperinsulinaemia and hyperglycaemia with cardiovascular disease in individuals without type 2 diabetes should provide insights into the mechanisms linking type 2 diabetes to increased cardiovascular disease risk.

Several studies have demonstrated that different measures of glucose or insulin metabolism or homeostasis are related to future CHD or stroke risk in individuals without diabetes [8–14], but few have compared the magnitude and independence of associations with markers that reflect insulin resistance and those that reflect reduced insulin secretion within the same study. It is therefore difficult to determine from current research whether the association between diabetes and cardiovascular disease is primarily driven by insulin resistance–related factors, insulin secretion–related factors, or both. Differences in associations across different studies are difficult to interpret since these might reflect different study protocols.

Further, few studies have explored these associations in women or in older individuals. For example, in two meta-analyses of the associations of glucose (fasting or post-load) with CHD or cardiovascular disease (including CHD, stroke, and other vascular outcomes) in individuals without diabetes, one reported that the vast majority of studies were of middle-aged men, with only 4% of the participants (2% of events) contributing to the pooled estimates being female [10]. In the second (more recent) meta-analysis this proportion had increased, but remained low with just 17% of participants (15% of events) being female [8]. In the Asia Pacific Collaboration (a meta-analysis of cohort studies from the Asia Pacific region), which included studies of the association of fasting glucose with CHD and stroke irrespective of whether participants with diabetes were excluded or not, 43% of the participants were female but less than one-third of the CHD events were in females [15]. In all three of these studies the mean age at baseline assessment of study participants was under 60 y in over 90% of the included studies; in the Asia Pacific Collaboration the mean age at baseline was particularly young at just 47 y across all studies. Similarly, in a recent meta-analysis of the association of insulin with cardiovascular disease just 11% of the cases were female and the mean age of participants was less than 60 y in all of the included studies [9].

There are several reasons why it is important to establish these associations in older women. CHD and stroke occur most commonly in those aged 60 y or over, and together occur equally in women and men. One cannot assume that associations of glucose and insulin metabolism with cardiovascular disease will be similar in women and men. Both the increased absolute and relative risk of CHD in individuals with diabetes is greater for women than men [16], suggesting that there are gender differences in the mechanism underlying this association.

The aim of this study was to examine the associations of fasting insulin, glucose, and glycated haemoglobin (HbA1c) with CHD and stroke in older women without diabetes (defined as no clinical diagnosis and a fasting glucose less than 7 mmol/l). This work will add to current research by directly comparing these markers of insulin resistance (fasting insulin) and insulin secretion (fasting glucose and HbA1c) within the same study population and by exploring these associations in older women.

## Methods

### Participants

Full details of the selection of participants and measurements have been previously reported [17]. Women aged 60–79 y were randomly selected from general practitioner lists in 23 British towns. A total of 4,286 women (60% of those invited) participated, and baseline data (self-completed questionnaire, research nurse interview, physical examination, and medical record review) were collected between April 1999 and March 2001. Over 99% of the participants were described by the examining nurse as “white,” with this ethnic distribution and the social class distribution of the participants in this cohort matching that of all British women of the same age based on census data from 1991 and 2001 [17]. For the present analyses we excluded all women with a medical diagnosis of diabetes or who had a fasting glucose of ≥ 7.0 mmol/l (*n* = 377), and all women who had a medical diagnosis of CHD or stroke (an additional *n* = 663), which left 3,246 women who fulfilled the eligibility criteria for the current study. These women have been followed up over a median of 4.6 y, to December 2004, by a detailed review of their medical records, conducted every 2 y, to identify nonfatal CVD events and, by flagging with the National Health Service Central Register (NHSCR), to obtain mortality data. There was no loss to follow-up for this linkage to vital status and medical records.

### Measurements

Venous blood samples were taken after a minimum 8 h fast (verified by the research nurse). Women who had blood samples taken in the morning had fasted overnight (i.e., for 10–12 h), whereas those who had samples taken later in the day had fasted for shorter periods (8–9 h) on average. There were no differences in any of the associations examined here when we stratified the sample according to whether the women had fasted overnight or not. Adjustment of our findings for time at which blood samples were taken did not affect any results.

Plasma glucose was measured by a glucose oxidase Trinder method [18] using a Hitachi Modular analyser. Serum insulin was measured using an ELISA assay that does not cross react with proinsulin [19]. HbA1c was measured on fasting whole blood using the Drew Hb Gold instrument, which is a High Performance Liquid Chromatography (http://www.drew-scientific.com/). We used the HOMA calculator (Oxford Centre for Diabetes, Endocrinology & Metabolism, Diabetes Trials Unit, http://www.dtu.ox.ac.uk/) to compute a measure of insulin sensitivity (HOMA-S) using fasting glucose and insulin [20]. This method of calculating HOMA uses a corrected formula that is believed to be a more accurate reflection of insulin sensitivity than the older method [20]. For HOMA-S higher values indicate less insulin resistance and are potentially less harmful to health. By contrast higher levels of fasting insulin are related to greater insulin resistance and are potentially harmful to cardiovascular health.

Incident CHD and stroke were the study outcomes. Incident cases of CHD (in those without baseline CHD) were defined as any of CHD death (ICD-10 codes I20-I25, I51.6) or a nonfatal myocardial infarction, angina diagnosis or coronary artery bypass, or angioplasty identified in the follow-up medical record reviews. Incident cases of stroke (in those without baseline stroke) were defined as either stroke death (ICD-10 codes I60-I69, G45) or occurrence of a nonfatal stroke identified in the follow-up medical record reviews.

We examined whether associations were independent of potential confounding factors (life course socioeconomic position [SEP], smoking, and physical activity) and whether they were independent of other components of the metabolic syndrome (high density lipoprotein cholesterol, triglyceride levels, systolic blood pressure, body mass index, and waist-to-hip circumference), which we did not consider to be confounding factors but which may provide insights into the mechanisms by determining whether any associations were mediated by other these relative risk factors.

High-density lipoprotein cholesterol (HDL-c) and triglyceride levels were measured, on fasting venous blood samples, using a Hitachi 747 automated analyser and reagents supplied by Roche Diagnostics (http://www.roche.com/div_diag.htm). Low-density lipoprotein cholesterol (LDL-c) was calculated using the Friedwald equation (LDL-c = total cholesterol − [HDL-c + triglycerides × 0.45]) [21]. Standard examination procedures were used to assess blood pressure, height, weight, and waist and hip circumference as previously described [17]. Data on childhood and adulthood SEP were obtained from baseline questionnaires and combined into a life-course SEP score, as previously described [21], which indicated the number of adverse life-course SEP from 0/1 through to 9/10. Information on smoking (categorised as never, past, and current) and physical activity (a frequency and duration questionnaire used to estimate hours spent in moderate or vigorous activity per week) was obtained from the baseline questionnaires and research nurse interviews.

### Study Ethics

Informed consent was obtained from the women to examine their medical records and link them to the NHSCR. Both local and multicentre ethics committees' approvals were obtained for the study.

### Statistical Analyses

Fasting insulin, glucose, HOMA-S, and triglyceride levels all had positively skewed distributions and therefore log-transformed values were used in the estimation of correlation coefficients and in the regression models. Pairwise Pearson correlation coefficients were estimated between fasting glucose, insulin, HOMA-S, HbA1c, and other continuous variables. In this population most of the strokes are likely to be ischaemic and therefore have a similar aetiology to CHD [23]. However, for all models we examined associations with CHD only, stroke only, and a combined outcome of CHD and stroke. We examined whether there was any evidence of heterogeneity between associations with these three different outcomes using a *z*-statistic. There was no evidence of heterogeneity for the different outcomes with any of the exposures in any models (all *p*-values >0.7).

Cox proportional hazard models were used to examine the prospective associations of both exposures and potential confounding factors with incident CHD and stroke (fatal and nonfatal). In the Cox proportional hazards models, the participant's age was the time axis, and risk was assessed from the date of baseline examination for each woman. Contributions to risk were censored at the date of first outcome event of interest, death from any other cause, or the end of the follow-up period (31 December 2004) for those who remained alive and free of CHD or stroke. In order to compare the magnitudes of the associations of each exposure (fasting glucose, insulin, and HbA1c) with the outcomes, we estimated effects (hazard ratios) per standard deviation (SD) of each exposure, using the SD of the log-transformed values of fasting insulin and glucose. Despite being censored at its upper end (7 mmol/l), the distribution of log-transformed values of fasting glucose in those women included in our analyses was approximately normal.

The nature of associations (linear and nonlinear) were examined in two ways. First, we examined associations of outcomes with fifths of the distribution of each exposure, and computed likelihood ratio tests to compare a model with these five categories entered as four indicator (dummy) variables (nonlinear model) to a model with the five categories entered as one variable—an ordered categorical variable (linear model). Second, we tested for statistical evidence of quadratic or higher power curvilinear associations by using each exposure as a continuous variable (per SD change) and computing a likelihood ratio test to compare each of a series of models that all contained the first-order term and additionally second-, third-, or fourth-order terms, with a model including only the first-order term. The advantage of the first approach is that it examines a general nonlinear association without specifying the particular form of this nonlinear association. The second approach tests specific curvilinear associations and produces a more specific description of any nonlinear association.

For several of the variables included in the analyses there were small amounts of missing data, varying from 0% to 13% for any individual variable (see Tables 1 and 2). Missing data resulted from a number of different reasons, including participant's nonresponse to specific questions, failure of participant to complete parts of the physical examination, failure of participant to provide a blood sample or to provide sufficient blood for all of the tests we proposed doing in this cohort study (we attempted to obtain 20 ml of blood from all participants), and inability of the laboratory to complete a particular assay on a particular sample.

**Table 1:**
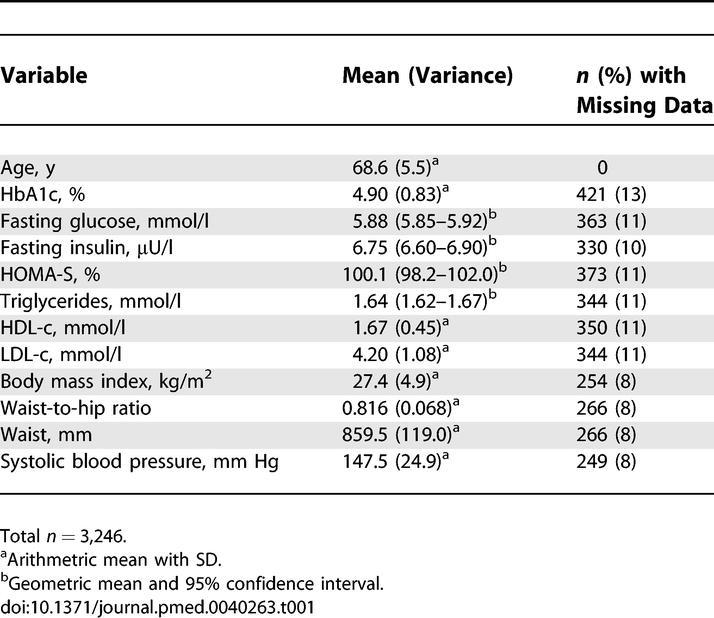
Baseline Characteristics of Women Aged 60–79 Years and Free of Diabetes, CHD, and Stroke: Continuous Variables

**Table 2:**
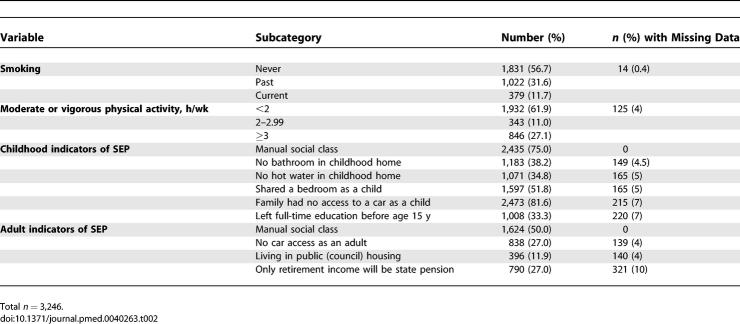
Baseline Characteristics of Women Aged 60–79 Years and Free of Diabetes, CHD, and Stroke: Categorical Variables

For the Cox proportional hazards models we used a multiple multivariate imputation, using all variables included in any analyses and the log of survival time for Cox models, to impute a distribution of missing values for those variables with some missing data with switching regression [24]. We carried out 20 cycles of regression switching, and generated ten imputation datasets. This approach creates a number of copies of the data (in this case we generated ten copies) each of which has values that are missing imputed with an appropriate level of randomness using chained equations [24]. The results are obtained by averaging across the results from each of these datasets using Rubin's rules, and the procedure takes account of uncertainty in the imputation as well as uncertainty due to random variation (as undertaken in all multivariable analyses) [24]. This method assumes that data are either “missing completely at random” or are “missing at random,” but are not “missing not at random” (i.e., it assumes that the probability of missing data does not depend on the outcome of interest). Although it is never possible to test this assumption, in this particular case it seems unlikely that the probability that a woman had missing information on her fasting values of glucose, insulin, or HbA1c, or the probability that she had missing data on some of the other covariables that were missing, was dependent upon her risk of experiencing a future CHD or stroke event. To further explore this possibility we also undertook all analyses on the complete dataset subsample. The results from the complete data subset analyses (*n* = 2,154 [66% of the 3,246 women included in the study]) were essentially the same as those obtained by combining results from the multiple imputation datasets, but were less precisely estimated. In this paper we present results from the multivariate multiple imputation models. Complete dataset subsample analyses are available in Table S1. All analyses were conducted using Stata version 9.2 (Stata, http://www.stata.com/).

## Results

Tables 1 and 2 show the baseline characteristics of the 3,246 women included in our analyses. Table 3 shows the pairwise correlations between continuously measured traits. There was near perfect inverse correlation between fasting insulin and HOMA-S and all associations in the Cox models were of the same magnitude (though inverse for HOMA-S) whichever of fasting insulin or HOMA-S was used, and therefore only associations with fasting insulin are presented. There were modest correlations between fasting glucose, insulin and HbA1c, and also modest correlations between each of these and most other continuous traits. The two exceptions were systolic blood pressure and LDL-c, which had only weak or no correlations with other variables. Waist circumference was strongly correlated with waist-to-hip ratio, and the magnitude of correlations between waist circumference with other metabolic and vascular measures were very similar to those presented in Table 3 for waist-to-hip ratio (unpublished data).

**Table 3:**
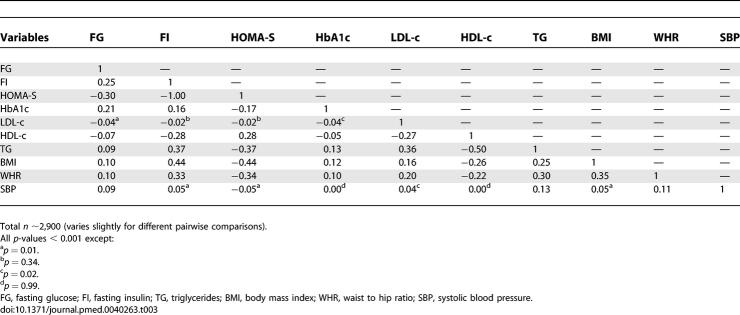
Pairwise Correlations between Continuous Variables in Women Aged 60–79 Years and without Diabetes

Of the 3,246 women included in the analyses, 174 experienced an incident case of CHD- giving a rate of 12.3 per 1,000 women-years (95% CI 10.6 to 14.2); 52 experienced an incident case of stroke, giving a rate of 3.6 per 1,000 woman-years (95% CI 2.7 to 4.7); and 219 experienced an incident case of either CHD or stroke giving a rate of 15.5 per 1,000 woman-years (95% CI 13.6 to 17.7). Note the total with either CHD or stroke (219) is smaller than the sum of those experiencing a CHD event (174) or a stroke (52), because seven women experienced both a CHD event and a stroke during the follow-up period.

Table 4 shows the age-adjusted (age being the time scale in the proportional hazards models) associations of potential confounding factors with CHD or stroke risk. Most established risk factors were associated with CHD or stroke in this cohort: low socioeconomic position, smoking, physical inactivity, higher waist-to-hip ratios, LDL-c, triglycerides, and systolic blood pressure and lower HDL-c levels. Body mass index was not associated with CHD or stroke in this cohort.

**Table 4:**
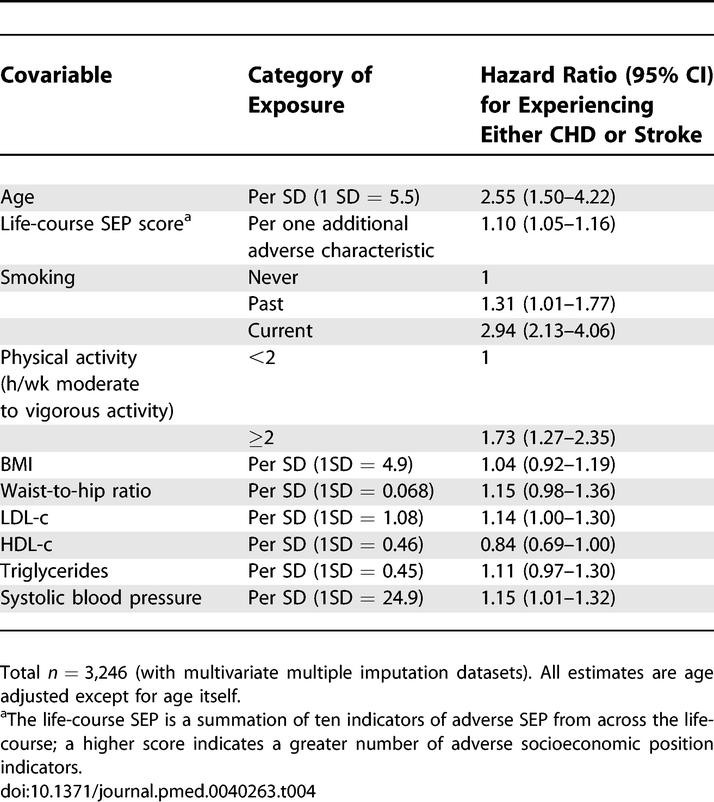
Age-Adjusted Associations of Covariable Factors with CHD or Stroke

Table 5 shows the multivariable associations of fasting glucose, insulin, and HbA1c with incident CHD and stroke. In these analyses only fasting insulin was associated with incident outcomes. The association of fasting insulin with incident outcomes was linear across the distribution with no statistical evidence from models with indicator variables or from higher-order curvilinear variables of nonlinear associations (all *p*-values >0.2). There was no evidence of linear or nonlinear associations of either fasting glucose or HbA1c with incident CHD or stroke (all *p*-values >0.3). The association of fasting insulin with incident outcomes attenuated with adjustment for potential confounders (model 2), with little further attenuation upon additional adjustment for other components of metabolic syndrome and LDL-c (model 3) or for fasting glucose and HbA1c (model 4). There was weak statistical evidence that the association of fasting insulin with incident CHD or stroke was greater than the association of either fasting glucose or HbA1c with this outcome (both *p* = 0.09). When we excluded the first year of follow-up in these analyses to minimise the possibility of reverse causality, the associations with CHD (*n* = 120 cases with first year excluded) and combined CHD and stroke (*n* = 157) were the essentially the same as those presented in Table 5.

**Table 5:**
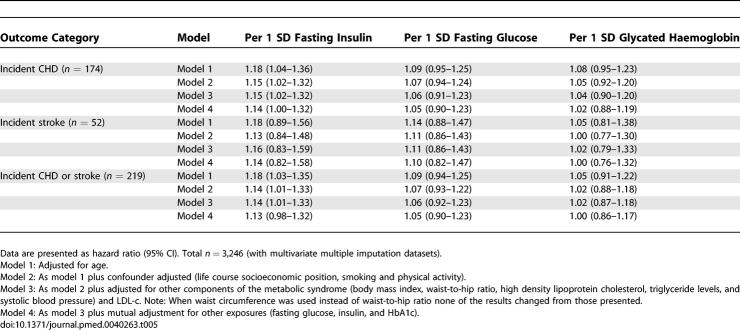
Multivariable Association of Fasting Glucose, Insulin, and Glycated Haemoglobin with Incident CHD and Stroke in Women Aged 60–79 Years and Free of Diabetes and Cardiovascular Disease at Baseline

We stratified the women into two age groups at baseline (60–69 y or 70–79 y) in order to determine whether any associations differed by age. Correlation coefficients between continuous variables were similar in each age group and there was no evidence that the associations of any of the exposures with incident outcomes differed by age group. All point estimates were similar for the two age groups, and *p*-values for interaction with age group were ≥ 0.7.

## Discussion

In this general population study of older British women who were free from clinically diagnosed diabetes and who had fasting glucose levels of less than 7 mmol/l and no clinical evidence of CHD or stroke at baseline, we found only modest correlations between fasting insulin, fasting glucose, and HbA1c levels. Only fasting insulin, but not glucose or HbA1c, was associated with incident CHD and stroke. This association remained with adjustment for potential confounding factors, other components of the metabolic syndrome, LDL-c, fasting glucose, and HbA1c. Few previous studies have directly compared the associations of fasting glucose, insulin, and glycated haemoglobin within the same study population, though several have compared each of these components singly within different studies.

Numerous studies have examined the association of fasting or post-load blood glucose levels in the nondiabetic range with CHD and/or stroke risk, but definitions and results have been inconsistent and few studies have examined associations in older women, a group at high risk for these outcomes. A meta-analysis of studies published up to 1996 (only 6% of participants were female and just 2% of events occurred in females) found that the association was consistent with the null hypothesis when the analyses were limited to categories with values lower than 7.8 mmol/l, though overall the authors concluded there was an exponential association between fasting glucose and CHD [10]. In a more recent meta-analysis (17% of participants were females, who accounted for 15% of events) the pooled relative risk for studies that strictly excluded individuals with baseline diabetes (diagnosed or with fasting glucose >7.0 mmol/l) was 1.26 (95% CI 1.11–1.43) comparing the highest to lowest glucose category and in studies that did not exclude individuals with high fasting glucose levels it was 1.48 (95% CI 1.25–1.75), with weak evidence that these two estimates differed from each other (*p* = 0.14) [8]. This recent meta-analysis also found a threshold effect of the association between fasting glucose and all cardiovascular diseases with no association in levels below 5.6 mmol/l [8]. Consistent with this latter meta-analysis in the Asia Pacific Collaboration, which included more females than previous meta-analyses (43% of participants, and ∼ 30% of CHD events and 50% of stroke events) there was a linear association of fasting glucose down to levels of 4.9 mmol/l for either CHD or stroke in both females and males [15]. However, participants in that study were considerably younger than in the present study (mean age 47 y) and there was some evidence that the association of fasting glucose with both CHD and stroke was weaker in those less than 60 y of age than in those older than 60 y [15]. For most of the studies included in the meta-analysis those with diabetes at baseline were not excluded, though existence of diabetes at baseline did not appear to be a major source of heterogeneity [15]. Taken together with our findings, these results suggest that fasting glucose may not be associated with future CHD or stroke risk across the range of normal glucose values. Our results suggest that in women aged 60–79 y with levels below the threshold of fasting glucose currently used to indicate diabetes (7 mmol/l), there appears to be no association of fasting glucose with CHD and stroke, though for the latter outcome, in particular, further larger studies are required.

Our finding that HbA1c is not associated with incident outcomes is consistent with findings from the Atherosclerosis Risk in Communities cohort, which found no overall linear association between HbA1c and incident CHD in nondiabetic individuals, though HbA1c was positively associated with incident CHD in those with a baseline value of HbA1c of greater than 4.6% in that study [25]. By contrast, the European Prospective Investigation into Cancer-Norfolk study found positive linear associations across the HbA1c distribution with incident cardiovascular disease (CHD, stroke, and other vascular and cardiac outcomes) and CHD events in women and men without diabetes [13]. In the main analyses in that study, individuals with baseline CVD were included and adjustment was made for prevalent disease. The positive association in that study may, therefore, be exaggerated by reverse causality. In a prospective case-control study nested within the Women's Health Study there was a positive association between HbA1c and incident CHD in women without diabetes in unadjusted analyses, but this attenuated completely to the null with adjustment for other CHD risk factor [26]. Thus, the overall evidence, while scant, does not suggest a strong association between HbA1c and CHD or all cardiovascular diseases in nondiabetic women.

Our findings of a positive linear association between fasting insulin and CHD and stroke events is consistent with findings from the Atherosclerosis Risk in Communities study, in which there was a positive linear association with CHD events that remained after adjustment for other CHD risk factors amongst women, but not amongst men [27]. A metaregression analysis of 17 prospective studies, primarily conducted in men and younger age groups than the current study, found a pooled relative risk of CHD per 50 pmol/l of insulin of 1.18 (95% CI 1.08–1.29) [9], which is consistent with our fully adjusted association with CHD (our results equate to 1.13 [95% CI 1.01–1.27] per 50 pmol/l of insulin). Overall the evidence suggests a modest positive association between fasting insulin and CHD events in women and men. Fasting insulin may exert its effect on cardiovascular risk via a direct impact on endothelial function [28,29].

### Study Limitations

In common with most epidemiological studies, we had just one baseline measurement of each exposure variable, and therefore our effect estimates may underestimate the true association if regression dilution bias is taken into account. Whilst oral glucose tolerance tests and eugylcaemic clamp studies are the gold standard for determining hyperglycaemia and insulin resistance they are difficult to conduct in large scale epidemiological studies [30]. Further, in routine clinical practice, assays of fasting blood samples to determine CHD risk are more feasible than these more complex methods. For women who were examined in the afternoon their fasting time (8–9 h) was shorter than the recommended 12 h. However, in stratified analyses all results were the same in those with an overnight fast and those who had fasted for a shorter time. Amongst participants in this study, consistent with other studies, mean fasting glucose and insulin levels fell slightly during the morning and then remained stable across time in the afternoon [31]. However, adjustment for time of day of blood sampling had no effect on any results presented here. We had too few incident cases of stroke for precise analyses and are unable to determine whether these were ischemic or haemorrhagic stroke. We found no statistical evidence of heterogeneity in effect between results from our three outcomes (CHD alone, stroke alone, or CHD and stroke combined), but would have limited power in this study to find heterogeneity if it existed because of the small number of stroke cases. In this population it is likely that the majority of strokes will be ischemic and as such have a similar pathophysiology to CHD [23,32], which justifies our combining these two outcomes in the main analyses. This is supported by the similarity of the point estimates for each outcome.

We defined our population as women without a clinical diagnosis of diabetes and in whom fasting glucose levels were less than 7 mmol/l, the current threshold for fasting glucose used to indicate diabetes. Had we been able to perform oral glucose tolerance tests on these women at baseline it is possible that some additional women would have also been excluded on the basis of elevated 2 h postload glucose. Our results are appropriately interpreted as suggesting that amongst older women with fasting glucose levels below 7 mmol/l fasting, insulin is a better predictor of future cardiovascular risk than fasting glucose or glycated haemoglobin.

Type 2 diabetes occurs when insulin resistance is combined with pancreatic β-cell dysfunction and hyperglycaemia results. There is debate about whether chronic hyperglycaemia in patients with diabetes is related to macrovascular complications, including CHD and stroke [33,34]. If chronic hyperglycaemia is important in the aetiology of CHD and stroke in individuals with type 2 diabetes, then it seems likely that his association would extend to those with high levels of fasting glucose and HbA1c (indicators of hyperglycaemia) in the nondiabetic population, but we found no such associations here. Thus, our findings suggest that insulin resistance may be the most important mechanism linking type 2 diabetes to CHD, and possibly stroke, in older women. However, this interpretation would require testing in large studies with oral glucose tolerance tests and insulin resistance measured by euglycaemic clamp studies, which are unlikely to be conducted for logistic reasons. Alternatively, as genomics expands it is likely that finely mapped genetic variants that affect insulin resistance but not hyperglycaemia and vice versa will be identified and robustly replicated. Once they are, such variants could be used as instrumental variables to determine the independent causal effects of insulin resistance and hyperglycaemia on cardiovascular disease risk [35]. If such studies confirm our findings, then from a public health point of view, interventions (both lifestyle and therapeutic) aimed at preventing insulin resistance might be more important than those aimed at preventing chronic hyperglycaemia. Understanding the relative contributions of insulin resistance and insulin secretion to the major consequences of type 2 diabetes, such as the development of CHD and stroke, is important for developing better-targeted therapeutics.

There is much debate about how best to identify individuals at high risk of CHD in clinical practice so that preventive interventions (changes in lifestyle and therapeutics) can be appropriately targeted to those at most risk [36,37]. It is now acknowledged that the Framingham equation varies in its ability to accurately determine risk in different populations determined by gender, age, and ethnicity [36,37]. There are also calls for improving prediction in all populations by including a greater number of (novel) CVD risk factors, though the best methods for assessing the additional predictive value of new variables is unclear [38,39]. Our findings suggest that including fasting insulin, but not fasting glucose or HbA1c, in CHD risk assessment of older women might improve prediction in clinical practice.

Our findings indicate that amongst older women without diabetes and with fasting glucose levels in the normal range, fasting insulin is a stronger predictor of CHD and stroke risk than are fasting glucose or HbA1c.

## Supporting Information

Table S1 Complete Case Multivariable Associations Listed are associations of fasting glucose, insulin, and glycated haemoglobin with incident coronary heart disease and stroke in women aged 60–79 y and free of diabetes and cardiovascular disease at baseline. Complete data subset *n* = 2,154.
(27 KB DOC)

## Abbreviations

CHD: coronary heart disease
CI: confidence interval
HbA1c: glycated haemoglobin
HDL-c: high-density lipoprotein cholesterol
HOMA-S: homeostasis model assessment (of insulin sensitivity)
LDL-c: low-density lipoprotein cholesterol
NHSCR: National Health Service Central Register
SD: standard deviation
SEP: socioeconomic position
