# Supplementary material for: Independent Associations of Fasting Insulin, Glucose, and Glycated Haemoglobin with Stroke and Coronary Heart Disease in Older Women
Source: PLoS Med. 2007 Aug 28;4(8):e263. doi: 10.1371/journal.pmed.0040263 (PMC1952205; doi:10.1371/journal.pmed.0040263)
Supplement: Table S1 — Listed are associations of fasting glucose, insulin, and glycated haemoglobin with incident coronary heart disease and stroke in women aged 60–79 y and free of diabetes and cardiovascular disease at baseline. Complete data subset n = 2,154. (27 KB DOC) [file pmed.0040263.st001.doc]

## Supplementary Web-table

Table W1: Multivariable association of fasting glucose, insulin and glycated haemoglobin with incident coronary heart disease and stroke in women aged 60-79 years and free of diabetes and cardiovascular disease at baseline.

**N = 2154** (complete data subset)

This is equivalent to table 4 in the main paper but here the analyses are done on only those women with complete data on any variables used in any model. The table presented in the paper is done by combining 10 multiple imputation datasets, in which missing data for any variable was imputed using multivariable imputation with chained equations (1).

|  | **Hazard ratio (95%CI)** | | |
| --- | --- | --- | --- |
|  | Per 1SD fasting insulin | Per 1SD fasting glucose | Per 1SD glycated haemoglobin |
| Outcome = Incident CHD (N = 129 cases) | | | |
| Model 1 | 1.26 (1.07, 1.51) | 1.10 (0.90, 1.32) | 1.09 (0.94, 1.27) |
| Model 2 | 1.20 (1.00, 1.45) | 1.09 (0.89, 1.33) | 1.04 (0.87, 1.24) |
| Model 3 | 1.17 (0.96, 1.43) | 1.09 (0.89, 1.33) | 1.02 (0.86, 1.22) |
| Model 4 | 1.15 (0.93, 1.41) | 1.08 (0.89, 1.32) | 1.00 (0.84, 1.21) |
| Outcome = Incident stroke (N = 33 cases) | | | |
| Model 1 | 1.19 (0.85, 1.67) | 1.14 (0.78, 1.67) | 1.07 (0.84, 1.36) |
| Model 2 | 1.13 (0.81, 1.60) | 1.11 (0.75, 1.63) | 1.02 (0.77, 1.33) |
| Model 3 | 1.10 (0.75, 1.58) | 1.09 (0.73, 1.60) | 1.02 (0.77, 1.33) |
| Model 4 | 1.08 (0.74, 1.56) | 1.08 (0.73, 1.59) | 1.00 (0.75, 1.32) |
| Outcome = Incident CHD or stroke (N = 152 cases) | | | |
| Model 1 | 1.27 (1.10, 1.51) | 1.09 (0.90, 1.29) | 1.09 (0.96, 1.25) |
| Model 2 | 1.23 (1.05, 1.44) | 1.08 (0.90, 1.29) | 1.05 (0.91, 1.23) |
| Model 3 | 1.15 (0.96, 1.36) | 1.07 (0.90, 1.28) | 1.04 (0.89, 1.21) |
| Model 4 | 1.12 (0.94, 1.34) | 1.07 (0.90, 1.28) | 1.02 (0.86, 1.19) |

Model 1: adjusted for age

Model 2: as model 1 plus confounder adjusted (life course socioeconomic position, smoking and physical activity)

Model 3: as model 2 plus adjusted for other components of the metabolic syndrome (body mass index, waist to hip ratio, high density lipoprotein cholesterol, triglyceride levels, systolic blood pressure) and LDLc. NB: when waist circumference was used instead of waist:hip ratio none of the results changed from those presented.

Model 4: as model 3 plus mutual adjustment for other exposures (fasting glucose, insulin and HbA1c)

**Reference**

1. Royston P (2004). Multiple imputation of missing values. *Stata Journal* 2004;**4**:227-241
